# Non-coding rnas in Turner syndrome: a systematic review

**DOI:** 10.1590/1984-0462/2025/43/2024029

**Published:** 2024-11-29

**Authors:** Júlio César Carvalho de Oliveira, Eldevan da Silva Barbosa, Nathaniel Batista Silva, Thaís da Conceição Silva, Ana Gabrielly de Melo Matos, Jaqueline Diniz Pinho

**Affiliations:** a Universidade Estadual do Maranhão, Zé Doca, MA, Brazil.

**Keywords:** Biomarkers, Genetic syndromes, Karyotype, Non-coding RNA, Biomarcadores, Síndromes genéticas, Cariótipo, RNAs não codificantes

## Abstract

**Objective:**

The aim of this study was to summarize the main findings of non-coding RNA (ncRNAs) in Turner syndrome (TS), correlating these biomolecules with the clinical manifestations in affected patients.

**Data source:**

Searches were conducted in the databases of the United States National Library of Medicine (PubMed), Scientific Electronic Library Online (SciELO), and ScienceDirect, covering original English articles published from 2014 to 2023. Descriptors used included "lncRNAs and Turner Syndrome," "miRNAs and Turner Syndrome," and "circRNAs and Turner Syndrome." The studies that were included addressed the role of ncRNAs in the clinical characteristics of patients with TS. Exclusion criteria comprised texts in abstracts, reports, reviews, and monographs.

**Data synthesis:**

We identified 147 studies, of which seven were included. In the analysis of microRNAs, miR-486-5p and miR-320a stood out, being associated with ovarian development; miR-126-3p and miR-126-5p were related to greater aortic stiffness. Regarding long non-coding RNAs, the downregulation of XIST indicated dysfunctions in X chromosome inactivation. Concerning circular RNAs, circPPP2R3B, circCSF2RA, and circPCTN were related to immunological functions, while circ_0090421, circ_0090392, and circ_0089945 were linked to cardiac development.

**Conclusions:**

The data from these studies demonstrate that these biomolecules play crucial roles in processes related to specific characteristics observed in TS patients. Besides being suggested as potential biomarkers, they may be useful in clinical practice.

## RESUMO

**Objetivo:**

Resumir as principais descobertas dos RNA não codificantes (ncRNAs) na síndrome de Turner (ST), correlacionando essas biomoléculas com as manifestações clínicas observadas nos pacientes afetados.

**Fontes de dados:**

Foram realizadas pesquisas nas bases de dados da Biblioteca Nacional de Medicina dos Estados Unidos (PubMed), Biblioteca Científica Eletrônica Online (SciELO) e ScienceDirect, abrangendo artigos originais em inglês publicados de 2014 a 2023. Os descritores utilizados foram: "lncRNAs e síndrome de Turner", "miRNAs e síndrome de Turner" e "circRNAs e síndrome de Turner". Os estudos incluídos abordaram o papel dos ncRNA nas características clínicas dos pacientes com ST. Os critérios de exclusão compreenderam textos de resumos, relatórios, resenhas e monografias.

**Síntese dos dados:**

Identificamos 147 estudos, dos quais sete foram incluídos no estudo. Na análise dos microRNAs, o miR-486-5p e o miR-320a se destacaram, estando associados ao desenvolvimento ovariano; o miR-126-3p e o miR-126-5p foram relacionados a uma maior rigidez aórtica. Com relação aos RNAs, a regulação negativa do XIST indicou disfunções na inativação do cromossomo X. Quanto aos circRNA, circPPP2R3B, circCSF2RA e circPCTN foram associados a funções imunológicas, enquanto circ_0090421, circ_0090392 e circ_0089945 foram vinculados ao desenvolvimento cardíaco.

**Conclusões:**

Os dados desses estudos demonstram que essas biomoléculas desempenham papéis cruciais em processos relacionados a características específicas observadas em pacientes com ST. Além de serem sugeridos como biomarcadores potenciais, os quais podem ser úteis na prática clínica.

## INTRODUCTION

Turner syndrome (TS), also known as X monosomy, was initially described by Henry Turner in 1938. However, it is important to highlight that a previously published case in 1930 by the German pediatrician Otto Ullrich concerning an eight-year-old girl with suggestive signs of TS played a crucial role in the initial characterization of this syndrome.^1,2^ Subsequently, Henry Ford clarified the cytogenetic basis, and Malcolm Ferguson-Smith suggested the presence of genes on the short arms of the sex chromosomes that would determine the TS phenotype.^3^

TS is a condition that exclusively affects the female biological sex, characterized by the total or partial loss of an X chromosome as its main feature. Approximately 2% of pregnancies are affected by this genetic condition; however, in 99% of cases, it results in spontaneous abortion. It is also noted that the average age for diagnosis is 15 years; however, many women with TS are not diagnosed until adulthood. Additionally, it is important to recognize that the life expectancy of those affected is lower than that of the general population.^4^

These patients may manifest various congenital and acquired phenotypic characteristics, including cardiovascular and renal problems, hearing impairment, hypertension, osteoporosis, and obesity, along with an increased incidence of autoimmune diseases such as thyroid disorders, vitiligo, and alopecia.^5,6^ There can be significant diversity in the frequency and intensity of dysmorphic signs in each patient, such as a short and/or webbed neck, broad shield-like chest, cubitus valgus, micrognathia, increased intermaxillary distance, hypoplastic nipples, low hairline, prominent and low-set ears, hyperconvex nails, strabismus, multiple pigmented nevi, eyelid ptosis, and epicanthic folds, among others.^5,7^

In addition to the signs above, many individuals with this syndrome experience psychosocial issues. There are reports of self-esteem issues, primarily due to short stature eight and delayed puberty development.^8,9^ The existence of a wide variety of signs and symptoms, coupled with the magnitude of these manifestations, can also have serious consequences on the psychological and social well-being of individuals with TS.^7^

In most cases, TS can be characterized by a karyotype of 45, X while mosaic patterns may be observed in approximately 30–40% of cases (45,X/46,XX and 45,X/46,XY). Alternatively, structural defects of the X chromosome may be present, such as deletions, isochromosomes of the long arm, or ring chromosomes.^10^ The phenotype manifested in TS is not exclusively determined by the simple genetic dosage resulting from X chromosome monosomy. The origin of specific comorbidities associated with TS may be influenced by a complex interplay between genes and transcriptional and epigenetic factors impacting gene expression throughout the genome.^11^

Therefore, the importance of epigenetic markers in understanding the pathophysiology of TS is notable, given that these modifications directly affect various genes on the X chromosome. Among the epigenetic markers, non-coding protein RNAs (ncRNAs) are prominent.^11^

Non-coding RNAs (ncRNAs) constitute 98% of the entire human genome.^12^ Despite lacking the potential for protein coding, they influence the expression of other genes through various mechanisms.^13^ Based on their function, ncRNAs are classified into two categories:

1. Maintenance ncRNAs (rRNAs, tRNAs, snRNAs, snoRNAs, TERC, tRF, and tiRNA) and
2. Regulatory ncRNAs (miRNAs, siRNAs, piRNAs, eRNAs, long non-coding RNAs (lncRNAs), circular RNAs (circRNAs), and Y RNAs).^14,15^

These biomolecules control gene expression at epigenetic, transcriptional, and post-transcriptional levels. The most well-known and extensively studied ncRNAs are miRNAs, which have approximately 20 nucleotides in their sequence.^16^

ncRNAs are extensively studied in various pathological conditions, such as cardiovascular diseases,^17^ neurological disorders,^18^ and malignant neoplasms.^19^ It is also essential to consider the relationship of these biomolecules with genetic syndromes, such as Down syndrome^20^ and Klinefelter syndrome.^21^ In Sjögren's syndrome, miR-146a has been suggested as a potential molecule involved in the early stages of the disease.^22^

However, few studies relate these biomolecules to the clinical features of affected patients in TS. In this context, the present review aims to summarize the main findings of ncRNAs in TS, correlating them with their clinical relevance and considering using these biomarkers to improve the quality of life for individuals with this syndrome.

## METHOD

This systematic literature review was conducted following the guidelines of PRISMA (Preferred Reporting Items for Systematic Reviews and Meta-analyses). The study was properly registered in PROSPERO (International Prospective Register of Systematic Reviews) under the registration number CRD42024498831. The review was done by consulting databases such as the US National Library of Medicine (PubMed), ScienceDirect, Scopus, and SciELO. Notably, studies lacking relevant information within the scope outlined for this work, as well as book chapters, reviews, and conference abstracts, were excluded from the analysis.

For the search and selection of articles, the descriptors "lncRNAs and Turner Syndrome," "miRNAs and Turner Syndrome," and "circRNAs and Turner Syndrome" were employed. The initial screening process involved the analysis of titles and abstracts. Studies that appeared potentially eligible were then subjected to a thorough assessment by two independent reviewers to evaluate compliance with predefined eligibility criteria. In cases of disagreement, a third reviewer conducted additional analysis to reach a consensus with the previous assessments.

Data from included studies was synthesized carefully and systematically, with information extracted and presented narratively. This procedure ensured the integrity and methodological rigor throughout the literature review process.

We exclusively included original articles written in English and published between 2014 and 2023, focusing on the role of ncRNAs in the clinical characteristics of patients with TS. Information such as these was extracted: clinical characteristics of patients associated with ncRNAs, types of ncRNAs, karyotype of patients with TS, ncRNA expression, and types of biological samples/sample population. Exclusion criteria comprised texts in abstracts, reports, reviews, and monographs.

Regarding assessing the methodological quality of the included studies, researchers evaluated them independently using the Joanna Institute Critical Appraisal Tools (JBI) checklist (JBI, 2020). Each criterion, as shown in Table 1 ^17,23-28^, was classified as "yes," "no," "unclear," or "not applicable." The risk of bias classification was determined based on scores: 1–3 "yes" indicated a high risk of bias, 4–6 "yes" indicated moderate bias, and 7–8 indicated low bias risk.

**Table 1:** Evaluation of the quality of methods utilized in the included studies using the *Joanna Institute Critical Appraisal Tools*.

Reference	Q1	Q2	Q3	Q4	Q5	Q6	Q7	Q8	Q9	Total (Yes)	Trend level
Umair et al.,^25^	Yes	Yes	N/A	Yes	N/A	Yes	Yes	Yes	Yes	7	Low
Abu-Halima et al.,^17^	Yes	Yes	N/A	Yes	Yes	N/A	Yes	Yes	Yes	7	Low
Abu-Halima et al.,^24^	Yes	Yes	N/A	Yes	N/A	Yes	N/A	Yes	N/A	5	Moderate
Sun et al.,^23^	Yes	Yes	N/A	Yes	Yes	Yes	N/A	Yes	Yes	7	Low
Rajpathak et al.,^26^	Yes	Yes	N/A	Yes	N/A	Yes	N/A	Yes	Yes	6	Moderate
Johannsen et al.,^28^	Yes	Yes	N/A	Yes	N/A	Yes	N/A	Yes	Yes	6	Moderate
Luo et al.,^27^	Yes	Yes	N/A	Yes	N/A	Yes	Yes	Yes	Yes	7	Low

N/A: Not applicable; Q1: Is the review question clearly and explicitly stated?; Q2: Were the inclusion criteria appropriate for the review question?; Q3: Was the search strategy appropriate?; Q4: Were the sources and resources used to search for studies adequate?; Q5: Were the criteria for appraising studies appropriate?; Q6: Was critical appraisal conducted by two or more reviewers independently?; Q7: Were there methods to minimize errors in data extraction?; Q8: Were the methods used to combine studies appropriate?; Q9: Was the likelihood of publication bias assessed?

## RESULTS

**Figure 1:**
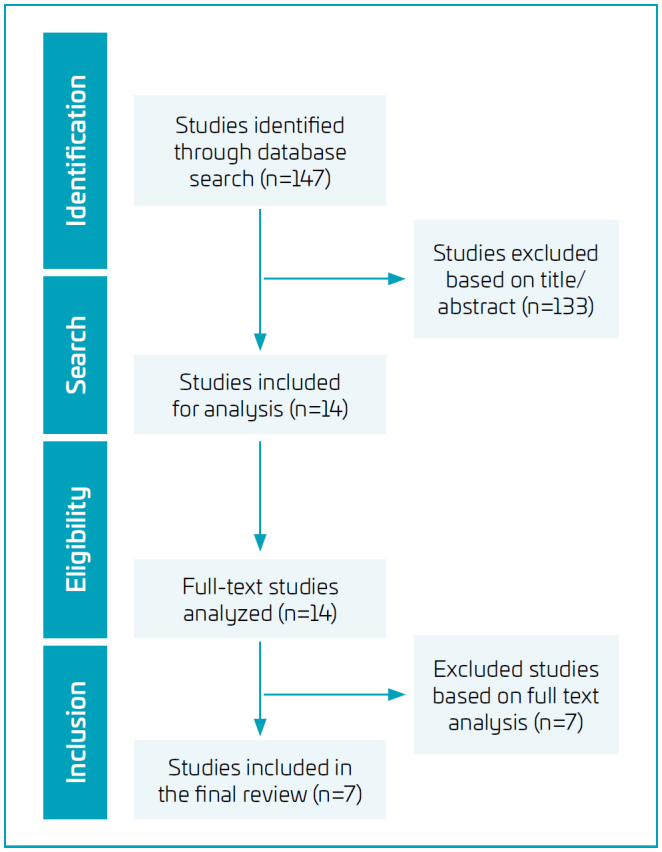
Flowchart depicting the selection and identification of studies, following the methodological steps outlined in the PRISMA guidelines.

During the research, a total of 147 studies were identified, and among these, 133 were excluded based on title/abstract screening. Consequently, 14 studies were selected for analysis. Of these, seven articles met the established inclusion criteria for the research (Figure 1).

Tables 2 ^17,23-25^, and 3^26-28^, list the classes of ncRNAs involved in clinical characteristics, types of ncRNAs, karyotype of patients with TS, types of expression, and characteristics of the sample and population. Four studies addressing the relationship of microRNAs in TS were found, with one focusing on lncRNAs and two articles exploring the clinical characteristics of TS patients concerning circRNAs.

**Table 2:** MicroRNAs involved in the clinical-pathological characteristics of Turner Syndrome.

Reference	Type of microRNAs	Types of biological samples/sample population	Clinical characteristics associated with ncRNAs	Karyotype of patients with TS	Additional information
**miRNA**					
Umair et al.,^25^	miR-4516 ↑	Urine and blood samples 65 Participants:18 patients with TS, 22 patients had premature ovarian insufficiency, and 25 controls.	Premature ovarian insufficiency	46,X,i(X)(q10),22pstk+[16]/45,X,22pstk+ 46,X,i(X)(q10) 45, X 46,X,i(X)(q10) 46,X,i(X)(q10)[24]/45,X[6]	Diagnostic biomarker
Abu-Halima et al.,^17^	miR-5695 ↑ miR-126-3p ↓	Blood samples 47 Participants: In which 33 patients presented the TS and 14 controls.	Coronary and aortic valve congenital disease	45, X Mosaic shape Structural chromosomal aberration	Diagnostic biomarker
Abu-Halima et al.,^24^	miR-126-3p ↑ miR-126-5p ↑	Blood samples 66 participants: 33 patients with ST and 33 control patients.	Aortic deformation and stiffness	45, X Mosaic shape Structural chromosomal aberration	Diagnostic biomarker
Sun et al.,^23^	miR-486-5p ↑ miR-320a ↑ let7a-5p↑ miR-10a-5p ↑	Plasma samples 132 patients: 50 participants with the TS, 50 control patients, and 32 patients recruited for other genetic tests.	Ovarian development and primordial germ cell survival	45, X	Circulating plasma miRNAs altered in TS

↑ Downregulated expression;

↓ Upregulated expression;

TS Turner Syndrome.

## DISCUSSION

### MicroRNAs and Turner syndrome

MicroRNAs (miRNAs) constitute a class of small ncRNAs conserved across diverse species, playing a pivotal role in the modulation of gene expression through the inhibition of translation or degradation of target messenger RNAs (mRNAs).^29^

The biogenesis of miRNAs commences in the cell nucleus, transcribed by RNA polymerase II, resulting in pri-microRNA. Subsequently, this is cleaved by the Drosha-DGCR8 RNase complex, forming a pre-microRNA. The pre-microRNA is transported to the cytoplasm with the assistance of the exportin-5/GTP61 complex, where it undergoes another cleavage by two proteins (Dicer and TRBP), giving rise to a mature miRNA comprising guide and passenger strands. One of these strands is then integrated into the RNA-induced silencing complex (RISC), which includes proteins such as Argonaute (AGO), forming a functional miRNA. The RISC complex targets the mRNA, leading to either mRNA degradation or the inhibition of translation. This intricate process of miRNA biogenesis plays a fundamental role in the post-transcriptional regulation of gene expression, influencing various cellular processes, from embryonic development to responses to environmental stimuli.^30^

About the works done with TS, we can mention that of Sun et al.,^23^ in which they compared the expression levels of miRNAs between women with karyotypes 45,X and 46,XX. They observed that among the 182 miRNAs analyzed, 85 showed significant alterations in TS, with notable changes in miR-486-5p, miR-320a, and let-7a-5p. MiR-486-5p and miR-320a were overexpressed in plasma samples, peripheral blood mononuclear cells (PBMCs), and fetal gonadal tissues in TS patients. Additionally, miR-320a and miR-486-5p are involved in the regulation of ovarian development.

Abu-Halima et al.,^17^ in turn, observed that among a group of 60 differentially expressed miRNAs in the blood of patients with TS, eight stood out (miR-374b-5p, miR-199a-5p, miR-340-3p, miR-125b-5p, miR-30e-3p, miR-126-3p, miR-5695, and miR-26b-5p). The overexpression of miR-5695 was higher in TS patients with coronary artery disease compared to those without coronary artery disease, while the low expression of miR-126-3p was lower in TS patients with congenital aortic valve disease compared to those without congenital valve disease.

Concerning cardiac conditions, another research carried out by Abu-Halima et al.^24^ revealed that miR-126-3p and miR-126-5p were found to be overexpressed in patients with TS when compared to healthy women. These microRNAs demonstrated high sensitivity and specificity in distinguishing between these two study groups. Cardiac anomalies in TS patients stand as the primary causes of mortality within this group, consequently leading to a reduced life expectancy.^31,32^ Additionally, in other studies involving syndromes where patients also present cardiac issues, microRNAs have been associated with these clinical characteristics.^33^

Premature ovarian insufficiency (POI) is another condition linked to the spectrum of malignancies in TS, as emphasized by Yoon et al.^32^ In this context, Umair et al.,^25^ utilizing non-invasive urine samples, observed an increased expression of miR-4516, miR-29a-3p, and miR-30b-5p in TS patients. Notably, the overexpression of miR-4516 was independent of the presence of POI. Hence, the data from this study demonstrated the feasibility of using non-invasive samples for TS diagnosis, with miR-29a and miR-30b identified as potential candidates and miR-4516 serving as a diagnostic marker for POI.

In conclusion, research on microRNAs in TS delves deeper into and provides significant information about the molecular alterations associated with this condition, thereby positioning them as potential biomarkers.

### lncRNAs and Turner syndrome

lncRNAs can be categorized based on their origin regions, which include:

1. lncRNA-sense,
2. lncRNA-antisense,
3. lncRNA-bidirectional,
4. lncRNA-intronic, and
5. lncRNA-intergenic.^34^

LncRNAs exhibit similarities to mRNAs, as most of them are transcribed by RNA polymerase II (Pol II) and undergo modifications at the RNA ends, such as adding the 5’ cap and polyadenylation at the 3’ end. They also undergo splicing.^35^

The findings from the analyses conducted by Rajpathak et al.^26^ indicate variations in the expression of five lncRNA transcripts between 45 X and 46 XX fibroblastic cells (Table 3). The majority of these transcripts exhibit negative regulation in 45 X cells. Among these lncRNAs, XIST stands out and is recognized for its role in X chromosome inactivation.^36^ The downregulation of XIST in 45 X cells suggests a possible dysfunction or alteration in X chromosome inactivation among patients with TS. This negative regulation is associated with bone differentiation, glucose metabolism, and gonadal development pathways.

**Table 3:** lncRNAs and circRNAs involved in the clinical-pathological characteristics of Turner Syndrome.

Reference	Type of lncRNAs/circRNAs	Types of biological samples/sample population	Clinical characteristics associated with ncRNAs	Karyotype of patients with TS	Additional information
**lncRNA**					
Rajpathak et al.,^26^	XIST ↓	Fibroblast cell lineage	Negative regulation in 45, X cells	45, X	Bone differentiation, glucose metabolism, and gonadal development pathways
**circRNA**					
Johannsen et al.,^28^	circVDAC3 ↑ circPCNT ↑ circCOL4A3BP ↑	Blood, muscle, and fat samples 115 participants: 33 had TS, and 22 with the Klinefelter syndrome, in addition to 16 male controls and 44 female controls	Increased risk of autoimmune diseases small immunological deficiencies 45, X	45, X	Differential expression in circRNAs Tissue-specific expression pattern
Luo et al.,^27^	circ_0090421 ↓ circ_0090392 ↓ circ_0089945 ↓ circ_0091070 ↓ circ_0090426 ↓ circ_0091293 ↓ circ_0090436 ↓ circ_0090390 ↓ circ_0090430 ↓ circ_0090434 ↓	Blood samples	Development of cardiac anomalies	45, X	Regulation of autosomal cardiac development genes via ceRNA network

↑ Downregulated expression;

↓ Upregulated expression;

TS Turner Syndrome.

The *XIST* gene's differential expression has been explored in other genetic syndromes, such as triple X syndrome^37^ and Down syndrome,^38^ which has been observed as a potential transcriptional silencer of chromosomes. The significant decrease in lncRNA XIST expression may, in part, contribute to the notably low risk of breast cancer observed in women with TS. Conversely, the elevated expression of this lncRNA in men with Klinefelter syndrome may be associated with an increased incidence of breast cancer in this specific group.^39,40^

Accordingly, studies have demonstrated that some lncRNAs can escape X chromosome inactivation, as shown in the research by Luo et al.,^27^ who identified certain lncRNAs that evade X chromosome inactivation, notably highlighting lnc-KDM5C. The host gene of lnc-KDM5C, *KDM5C*, is associated with cardiovascular development.

These data illustrate that certain lncRNAs, especially XIST and lnc-KDM5C, can evade X chromosome inactivation. They play a role in processes that give rise to specific conditions in individuals with TS and possibly in other genetic conditions as well.

### circRNAs and Turner syndrome

circRNAs, recently identified ncRNAs, consist of over 200 nucleotides forming a cyclic structure.^41^ These molecules are covalently closed and circularized, connecting the 3’ and 5’ ends through exon or intron circularization.^42^ In eukaryotes, spliceosomes perform their usual function of excising introns from mRNA, forming a linear RNA molecule. In contrast to this conventional splicing pattern, circRNA undergoes a distinctive process called back-splicing, where the 5’ and 3’ ends of the RNA molecule connect through a 3’–5’ phosphodiester linkage, resulting in a circular-like structure.^43^ This process is coordinated by RNA-binding proteins, transcription factors, *cis*-acting elements, and *trans*-acting factors, establishing a competitive relationship between conventional splicing and back-splicing.^44^

CircRNAs have been identified with diverse biological functions, including their ability to bind to miRNAs and proteins, regulate transcriptional and post-transcriptional levels, modulate parental gene expression, and even encode proteins. These circRNAs play crucial roles in physiological and pathological development across various organisms.^45^ Recent research has revealed a correlation between circRNA expression levels and pathological processes, ranging from tumor development to degenerative diseases, autoimmune conditions, and even DS.^46-49^

A study conducted by Johannsen et al.^28^ investigated the circRNA profile in women with TS (45,X; TS) and men with Klinefelter syndrome (47,XXY;KS) investigated (Table 3). The researchers observed differential expression of these biomolecules throughout the genome. Among the circRNAs identified in TS, circPPP2R3B, circCSF2RA, circPCTN, circTRABDA, circZNF292, circVDAC3, and circCOL4A3BP were highlighted by the authors. CircPPP2R3B showed expression in muscle samples from all karyotypes except for 45,X. CircCSF2RA, on the contrary, exhibited expression in blood and fat samples. CircPCTN, circTRABDA, and circZNF292 were associated with immune system pathways, particularly neutrophil functions, suggesting that these circRNAs may contribute to an increased risk of autoimmune diseases and mild immune deficiencies in women with TS. In the same study, it was observed that in TS, circVDAC3, circPCNT, and circCOL4A3BP are positively regulated and interact with the miRNAs miR-150-5p, miR-26b-5p, miR-520g-3p, and miR-520h.

The study conducted by Luo et al.^27^ identified several circRNAs with differential expression, including circ_0090421, circ_0090392, circ_0089945, circ_0091070, circ_0090426, circ_0091293, circ_0090436, circ_0090390, circ_0090430, and circ_0090434, which are associated with cardiac development. For instance, circ_0090421 has its host gene in *CDK16*, and circ_0090392 is in the *UBA*1 gene. These autosomal genes may regulate cardiac development genes through endogenous competition.

Consequently, these findings illustrate that the differential expression of circRNA could regulate mechanisms underlying TS. Studies on circRNAs present a promising field in biological research, offering valuable insights for comprehending genetic regulation in diverse contexts.

### Future perspectives

The prospects of utilizing ncRNAs for TS represent a promising and innovative area in medical research. ncRNAs, especially miRNAs and lncRNAs, have emerged as crucial regulators of cellular and molecular processes.^17,26^ These ncRNAs can play significant roles in gene regulation, cell differentiation, and organ development, as observed in the works of Abu-Halima et al.,^17^ Umair et al.,^25^ and Rajpathak et al.^26^

Investigating the expression profiles of ncRNAs in individuals with TS makes it possible to identify specific patterns associated with the condition. This can provide valuable insights into the pathophysiology of this syndrome, as described by Umair et al.^25^ and Abu-Halima et al.^17^ Thus, potentially guiding the development of more personalized and effective therapeutic approaches in the future.^19,50^

The ongoing advancements in sequencing technology and RNA analysis techniques offer opportunities to explore the potential of ncRNAs as biomarkers. This can enable earlier and more efficient diagnosis of anomalies resulting from X chromosome monosomy, leading to increasingly early interventions and therapies. This, in turn, will contribute to better management of challenges associated with the syndrome.^51^

It is necessary to explore the involvement of other ncRNAs, such as snoRNAs reported in Prader-Willi syndrome,^52,53^ and piRNAs, which have been reported in other syndromes like Rett syndrome.^54^ Additionally, conducting studies on the use of silencing techniques^55^ is essential, as these efforts can facilitate the identification of therapeutic targets and the development of personalized therapies.

Therefore, this review illustrates that ncRNAs play important roles in TS, such as the regulation of genes or pathways related to specific characteristics observed in these patients. Additionally, they are suggested as potential biomarkers in clinical practice.

## Data Availability

The database that originated the article is available with the corresponding author.

## References

[1] Turner HH (1938). A syndrome of infantilism, congenital webbed neck, and cubitus valgus. Endocrinology.

[2] Ullrich O (1930). Über typische Kombinationsbilder multipler Abartungen. Z. Kinder-Heilk.

[3] Ferguson-Smith MA, Boyd E, Ferguson-Smith ME, Pritchard JG, Yusuf AF, Gray B (1969). Isochromosome for long arm of Y chromosome in patient with Turner's syndrome and sex chromosome mosaicism (45,X-46,XYqi). J Med Genet.

[4] Finozzi R, Álvarez C (2022). Síndrome de Turner. Arch Pediatr Urug.

[5] Batch J (2002). Turner syndrome in childhood and adolescence. Best Pract Res Clin Endocrinol Metab.

[6] Rosenfeld RG (1992). Turner syndrome: a guide for physicians.

[7] Suzigan LZ, Silva RB, Maciel-Guerra AT (2005). Aspectos psicossociais da síndrome de Turner. Arq Bras Endocrinol Metabol.

[8] Rovet JF, Van Vliet G (2019). Growth hormone supplementation and psychosocial functioning to adult height in Turner syndrome: a questionnaire study of participants in the Canadian Randomized Trial. Front Endocrinol (Lausanne).

[9] Dörr HG, Bettendorf M, Binder G, Brämswig J, Hauffa BP, Holterhus PM (2019). Life situation of young women with Turner syndrome: results of a questionnaire-based study in Germany. Dtsch Med Wochenschr.

[10] Sybert VP, McCauley E (2004). Turner's syndrome. N Engl J Med.

[11] Viuff M, Skakkebaek A, Nielsen MM, Chang S, Gravholt CH (2019). Epigenetics and genomics in Turner syndrome. Am J Med Genet C Semin Med Genet.

[12] Santosh B, Varshney A, Yadava PK (2015). Noncoding RNAs: biological functions and applications. Cell Biochem Funct.

[13] Matsui M, Corey DR (2017). Noncoding RNAs as drug targets. Nat Rev Drug Discov.

[14] Taft RJ, Pang KC, Mercer TR, Dinger M, Mattick JS (2010). Non-coding RNAs: regulators of disease. J Pathol.

[15] Zhang P, Wu W, Chen Q, Chen M (2019). Non-Coding RNAs and their Integrated Networks. J Integr Bioinform.

[16] Mirna M, Paar V, Rezar R, Topf A, Eber M, Hoppe UC (2019). MicroRNAs in inflammatory heart diseases and sepsis-induced cardiac dysfunction: a potential scope for the future?. Cells.

[17] Abu-Halima M, Oberhoffer FS, El Rahman MA, Jung AM, Zemlin M, Rohrer TR (2020). Insights from circulating microRNAs in cardiovascular entities in turner syndrome patients. PloS One.

[18] Das T, Das TK, Khodarkovskaya A, Dash S (2021). Noncoding RNAs and their bioengineering applications for neurological diseases. Bioengineered.

[19] Toden S, Zumwalt TJ, Goel A (2021). Non-coding RNAs and potential therapeutic targeting in cancer. Biochim Biophys Acta Rev Cancer.

[20] Venegas-Zamora L, Bravo-Acuña F, Sigcho F, Gomez W, Bustamante-Salazar J, Pedrozo Z (2022). New molecular and organelle alterations linked to down syndrome heart disease. Front Genet.

[21] Cimino L, Salemi M, Cannarella R, Condorelli RA, Giurato G, Marchese G (2017). Decreased miRNA expression in Klinefelter syndrome. Sci Rep.

[22] Pauley KM, Stewart CM, Gauna AE, Dupre LC, Kuklani R, Chan AL (2011). Altered miR-146a expression in Sjögren's syndrome and its functional role in innate immunity. Eur J Imunol.

[23] Sun YX, Zhang YX, Zhang D, Xu CM, Chen SC, Zhang JY (2017). XCI-escaping gene KDM5C contributes to ovarian development via downregulating miR-320a. Hum Genet.

[24] Abu-Halima M, Oberhoffer FS, Wagner V, El Rahman MA, Jung AM, Zemlin M (2022). MicroRNA-126-3p/5p and aortic stiffness in patients with Turner syndrome. Children (Basel).

[25] Umair Z, Baek MO, Song J, An S, Chon SJ, Yoon MS (2022). MicroRNA-4516 in urinary exosomes as a biomarker of premature ovarian insufficiency. Cells.

[26] Rajpathak SN, Vellarikkal SK, Patowary A, Scaria V, Sivasubbu S, Deobagkar DD (2014). Human 45,X fibroblast transcriptome reveals distinct differentially expressed genes including long noncoding RNAs potentially associated with the pathophysiology of turner syndrome. PLoS One.

[27] Luo Y, Chen Y, Ge L, Zhou G, Chen Y, Zhu D (2023). Competing endogenous RNA network analysis of Turner syndrome patient-specific iPSC-derived cardiomyocytes reveals dysregulation of autosomal heart development genes by altered dosages of X-inactivation escaping noncoding RNAs. Stem Cell Res Ther.

[28] Johannsen EB, Just J, Viuff MH, Okholm TL, Pedersen SB, Lauritsen KM (2022). Sex chromosome aneuploidies give rise to changes in the circular RNA profile: a circular transcriptome-wide study of Turner and Klinefelter syndrome across different tissues. Front Genet.

[29] Correia MS, Gjorgjieva M, Dolicka D, Sobolewski C, Foti M (2019). Deciphering miRNAs’ Action through miRNA Editing. Int J Mol Sci.

[30] Zhang YL, Liu L, Peymanfar Y, Anderson P, Xian CJ (2021). Roles of MicroRNAs in osteogenesis or adipogenesis differentiation of bone marrow stromal progenitor cells. Int J Mol Sci.

[31] Schoemaker MJ, Swerdlow AJ, Higgins CD, Wright AF, Jacobs PA, United Kingdom Clinical Cytogenetics Group (2008). Mortality in women with turner syndrome in Great Britain: a national cohort study. J Clin Endocrinol Metab.

[32] Yoon SH, Kim GY, Choi GT, Do JT (2023). Organ abnormalities caused by Turner syndrome. Cells.

[33] Zbucka-Kretowska M, Niemira M, Paczkowska-Abdulsalam M, Bielska A, Szalkowska A, Parfieniuk E (2019). Prenatal circulating microRNA signatures of foetal Down syndrome. Sci Rep.

[34] Jarroux J, Morillon A, Pinskaya M (2017). History, discovery, and classification of lncRNAs. Adv Exp Med Biol.

[35] Bridges MC, Daulagala AC, Kourtidis A (2021). LNCcation: lncRNA localization and function. J Cell Biol.

[36] Li J, Ming Z, Yang L, Wang T, Liu G, Ma Q (2022). Long noncoding RNA XIST: Mechanisms for X chromosome inactivation, roles in sex-biased diseases, and therapeutic opportunities. Genes Dis.

[37] Nielsen MM, Trolle C, Vang S, Hornshøj H, Skakkebaek A, Hedegaard J (2020). Epigenetic and transcriptomic consequences of excess X-chromosome material in 47,XXX syndrome-A comparison with Turner syndrome and 46,XX females. Am J Med Genet C Semin Med Genet.

[38] Chiang JC, Jiang J, Newburger PE, Lawrence JB (2018). Trisomy silencing by XIST normalizes Down syndrome cell pathogenesis demonstrated for hematopoietic defects in vitro. Nat Commun.

[39] Swerdlow AJ, Schoemaker MJ, Higgins CD, Wright AF, Jacobs PA, UK Clinical Cytogenetics Group (2005). Cancer incidence and mortality in men with Klinefelter syndrome: a cohort study. J Natl Cancer Inst.

[40] Viuff MH, Stochholm K, Lin A, Berglund A, Juul S, Gravholt CH (2021). Cancer occurrence in Turner syndrome and the effect of sex hormone substitution therapy. Eur J Endocrinol.

[41] Tian H, Zhao L, Li H, Huang Y, Wang Y (2023). Circular RNA in retina: a potential biomarker and therapeutic target. Ophthalmic Res.

[42] Zhang W, Li L, Si Y, Shi Z, Zhu T, Zhuang S (2018). Identification of aberrant circular RNA expression and its potential clinical value in primary great saphenous vein varicosities. Biochem Biophys Res Commun.

[43] Malviya A, Bhuyan R (2023). The recent advancements in circRNA research: from biogenesis to therapeutic interventions. Pathol Res Pract.

[44] Ding C, Zhou Y (2023). Insights into circular RNAs: biogenesis, function and their regulatory roles in cardiovascular disease. J Cell Mol Med.

[45] Wang Q, Sun Y, Zhao Q, Wu W, Wang L, Miao Y (2022). Circular RNAs in pulmonary hypertension: emerging biological concepts and potential mechanism. Animal Model Exp Med.

[46] Sui W, Gan Q, Chang Y, Ou M, Chen J, Lin H (2020). Differential expression profile study and gene function analysis of maternal foetal-derived circRNA for screening for Down's syndrome. Exp Ther Med.

[47] Huang Y, Xue Q, Cheng C, Wang Y, Wang X, Chang J (2023). Circular RNA in autoimmune diseases: special emphasis on regulation mechanism in RA and SLE. J Pharm Pharmacol.

[48] Li F, Li PF, Hao XD (2023). Circular RNAs in ferroptosis: regulation mechanism and potential clinical application in disease. Front Pharmacol.

[49] Lin Z, Ji Y, Zhou J, Li G, Wu Y, Liu W (2023). Exosomal circRNAs in cancer: implications for therapy resistance and biomarkers. Cancer Lett.

[50] Traber GM, Yu AM (2023). RNAi-Based therapeutics and novel RNA bioengineering technologies. J Pharmacol Exp Ther.

[51] Gravholt CH, Viuff M, Just J, Sandahl K, Brun S, van der Velden J (2023). The changing face of Turner syndrome. Endocr Rev.

[52] Anderlid BM, Lundin J, Malmgren H, Lehtihet M, Nordgren A (2014). Small mosaic deletion encompassing the snoRNAs and SNURF-SNRPN results in an atypical Prader-Willi syndrome phenotype. Am J Med Genet A.

[53] Cassidy SB, Schwartz S, Miller JL, Driscoll DJ (2012). Prader-Willi syndrome. Genet Med.

[54] Saxena A, Tang D, Carninci P (2012). piRNAs warrant investigation in Rett syndrome: an omics perspective. Dis Markers.

[55] Gao L, Zhao Y, Wu H, Lin X, Guo F, Li J (2023). Polycystic ovary syndrome fuels cardiovascular inflammation and aggravates ischemic cardiac injury. Circulation.

